# Comparison of serum pools and oral fluid samples for detection of porcine circovirus type 2 by quantitative real-time PCR in finisher pigs

**DOI:** 10.1186/s40813-018-0079-4

**Published:** 2018-02-01

**Authors:** Gitte Blach Nielsen, Jens Peter Nielsen, John Haugegaard, Sanne Christiansen Leth, Lars E. Larsen, Charlotte Sonne Kristensen, Ken Steen Pedersen, Helle Stege, Charlotte K. Hjulsager, Hans Houe

**Affiliations:** 1 MSD Animal Health Nordic, Havneholmen 25, 1561 Copenhagen V, Denmark; 2 Department of Veterinary and Animal Sciences, University of Copenhagen, Grønnegårdsvej 2+8, 1870 Frederiksberg C, Denmark; 3 Porcus Veterinary Pig Practice, Ørbækvej 276, 5220 Odense, SØ Denmark; 4 National Veterinary Institute, Henrik Dams Allé, Bygning 205B, 2800 Kgs. Lyngby, Denmark; 5 SEGES Pig Research Centre, Vinkelvej 11, 8620 Kjellerup, Denmark; 6 Ø-vet A/S, Køberupvej 33, 4700 Næstved, Denmark

**Keywords:** Diagnostics, Finishers, Oral fluid, Pooling, Porcine circovirus type 2, Serum

## Abstract

**Background:**

Porcine circovirus type 2 (PCV2) diagnostics in live pigs often involves pooled serum and/or oral fluid samples for group-level determination of viral load by quantitative real-time polymerase chain reaction (qPCR). The purpose of the study was to compare the PCV2 viral load determined by qPCR of paired samples at the pen level of pools of sera (SP) from 4 to 5 pigs and the collective oral fluid (OF) from around 30 pigs corresponding to one rope put in the same pen. Pigs in pens of 2 finishing herds were sampled by cross-sectional (Herd 1) and cross-sectional with follow-up (Herd 2) study designs. In Herd 1, 50 sample pairs consisting of SP from 4 to 5 pigs and OF from around 23 pigs were collected. In Herd 2, 65 sample pairs consisting of 4 (SP) and around 30 (OF) pigs were collected 4 times at 3-week intervals.

**Results:**

A higher proportion of PCV2-positive pens (86% vs. 80% and 100% vs. 91%) and higher viral loads (mean difference: 2.10 and 1.83 log(10) PCV2 copies per ml) were found in OF versus SP in both herds. The OF cut-off value corresponding to a positive SP (>3 log(10) PCV2 copies per ml) was estimated to 6.5 and 7.36 log(10) PCV2 copies per ml for Herds 1 and 2, respectively. Significant correlations between SP and OF results were found in Herd 1 (rho = 0.69) and the first sampling in Herd 2 (rho = 0.39), but not for the subsequent consecutive 3 samplings in Herd 2.

**Conclusions:**

The proportion and viral loads of PCV2 positive pens were higher in collective OF (including up to 30 pigs) compared to SP (including 4–5 pigs) of the same pens. Also, OF seemed to detect the PCV2 infection earlier with OF values just below 6.5 (Herd 1) and 7.36 (Herd 2) log(10) being associated with a negative SP for the same pen. Nevertheless, a statistically significant correlation between SP and OF could not be found for all sampling time points, probably due to a high within-pen variation in individual pig viral load becoming very evident in SP of only four or five pigs. Consequently, the results imply that OF is well suited for detecting presence of PCV2 but less so for determining the specific viral load of pigs in a pen.

## Background

Porcine circovirus type 2 (PCV2), a circular, single-stranded, non-enveloped DNA virus has been demonstrated to be present in almost all commercial swine herds worldwide [1, 2]. PCV2 is the essential infectious agent involved in post-weaning multi-systemic wasting syndrome (PMWS) in pigs [3, 4]. The virus has been detected in serum and tissues (lymph nodes, lung, tonsil, kidney, liver, heart) and is shed in a variety of secretions (nasal, oral, fecal, urinary) [5–7]. In serum, individual viral loads above 7 log(10) PCV2 copies per ml serum have been associated with occurrence of clinical signs [8–10]. Currently, however, infection with PCV2 most commonly leads to subclinical infections that still negatively impact production parameters resulting in economic losses for the farmer [11]. Thus, the average daily gain has been found significantly reduced in pigs with viral loads as low as 4.3–5.3 log(10) PCV2 copies per ml serum [12].

Vaccination provides an effective tool to control PCV2 infections [13–17] but is an extra cost. Valid diagnostic tests enabling determination of the PCV2 viral load in a specific herd are therefore crucial when deciding whether or not to vaccinate. Furthermore, in cases of subclinical infections or infection with non-specific clinical signs, methods to confirm the diagnosis without euthanasia of pigs are highly preferable. For this purpose, virus detection in serum samples by quantitative real-time polymerase chain reaction (qPCR) has been widely used. To save laboratory costs in clinical swine practice, it has furthermore become increasingly popular to pool serum samples and then interpret the result as being representative for the age group sampled. Serum samples and nasal swabs have been suggested to be more suitable for evaluating PMWS status for a group of pigs than for individuals [9] and pooled samples could be a further development of this. However, it was later concluded that qPCR testing of pooled serum samples was not sufficiently reliable for diagnosis of PMWS at herd level but might be useful for determination of viral loads [18].

In the last 10 years, oral fluid sampling has gained growing interest as an even more cost and time-saving method while also considering animal welfare. The sampling procedure consists of hanging a cotton rope in a pen for the pigs to chew on, followed by wringing of the rope to release the oral fluid. The method was initially described in 2008 [19, 20], and the suitability of oral fluids for diagnosing different porcine pathogens by PCR such as PRRSV, swine influenza virus, foot-and-mouth disease virus, African and classical swine fever viruses as well as *Haemophilus parasuis* and *Streptococcus suis* has since been demonstrated [20–23]. Also for PCV2, qPCR of oral fluid has been proven valid for detection of infection [19, 24–26].

Some previous studies have compared detection and load of PCV2 by PCR in serum and oral fluid. A fair agreement between individual PCV2-positive serum and oral fluid samples (kappa = 0.24) but a poor agreement between pooled serum and oral fluid collected from pen-housed pigs (kappa = 0.001, 8–15 pigs per pen) has been found [27]. Another study reported that in 57 oral fluid samples of 3 PCV2-inoculated pens, 56 samples were PCV2-positive, whereas all 19 oral fluid samples in one pen of negative control pigs were PCV2-negative. Consequently, a sensitivity of 98% and a specificity of 100% of oral fluid were calculated [25, 28]. A very recent study found a higher proportion of PCV2-positive pens when PCR-analysis was done on oral fluid (11–23 pigs per pen) compared to pooled serum (2–4 pigs) and a relatively high, but non-significant, correlation (*r* = 0.76) between viral loads in the two sample types [29]. A similar correlation (*r* = 0.78) between viral loads in oral fluid (20–30 pigs per pen) and pooled serum (5 pigs) was found to be significant in another study [24]. One small experimental study reported a difference in the median viral loads between oral fluid and serum of around 1 log(10) based on 10 repeated samplings of the same pen [30]. A similar difference between mean viral loads in oral fluid and pooled serum samples at one sampling time point (including 40 pens) has been mentioned briefly elsewhere [29].

The inconclusive and limited number of studies regarding the association between viral loads in pooled serum and oral fluid at pen level and possible differences between PCR-assays require further elucidation by including additional herds/samples for the comparison. Moreover, since both oral fluid and pooled serum sampling are done with the purpose of diagnosing PCV2 infection in a group of pigs, determination of the oral fluid viral load corresponding to a positive serum pool is relevant for practical validation of oral fluid as a substitute for serum pools. Consequently, the objectives of this study were to: 1) determine the oral fluid viral load cut-off agreeing best with a PCV2-positive serum pool and 2) compare viral loads of PCV2 in serum pools and collective oral fluid from pigs in the same pen.

## Methods

### Herds

Serum and oral fluid samples were collected in 2 PCV2-infected finishing herds (Herd 1 and 2). Neither of the herds experienced clinical signs attributable to PCV2 infection (wasting, dyspnea or enlarged lymph nodes) [11]. Herd 1 was a conventional herd known to be seropositive for porcine reproductive and respiratory syndrome virus (PRRSv) and samples were collected in August 2010. Herd 2 was a specific-pathogen-free herd (free from *Mycoplasma hyopneumoniae*, *Actinobacillus pleuropneumoniae* type 2 + 6 + 12 and PRRSv) and samples were collected between September 2014 and July 2015 as a part of a larger field trial. None of the herds vaccinated against PCV2 prior to initiation of sampling, but in the field trial in Herd 2, half of the finishers were vaccinated during sample collection as a part of a PCV2 vaccine trial. However, only PCV2-qPCR results from the non-vaccinated group were included in the present study and an overview of the serum results have been presented briefly elsewhere [31].

### Sample size calculations

At the time of sampling in Herd 1 (August 2010), no previous studies had estimated the correlation coefficient between serum pools and oral fluid samples and the interest of the study was therefore to determine whether or not a correlation existed. Therefore, the sample size calculation was based on detecting a correlation coefficient equal to or higher than 0.4 at a significance level of 95% and a power of 0.8 corresponding to a sample size of 47 [32]. In Herd 2 (where the primary purpose was to detect a difference in feed conversion rate between vaccinated and control pigs), a sample size of 65 pens was estimated [31], which in terms of correlation between serum and oral fluid corresponded to detecting a significant correlation at a 95% level with a power of 0.8, if the correlation coefficient was equal to or higher than 0.34 [32].

### Study design

The study design in Herd 1 was cross-sectional with all samples collected from pigs of 3 different age groups in one day. The study design in Herd 2 was cross-sectional with follow-up consisting of totally 4 repeated samplings of the same pigs/pens at 3-week intervals. Serum and oral fluid were collected simultaneously at each sampling.

### Selection of study units

The study unit was the pen. Herd 1 had a total of 64 pens of which 50 were randomly (with age-stratification) selected for sampling [33]. In Herd 2, all pens with non-vaccinated finishers in 14 finishing batches were sampled, corresponding to a total number of 65 pens, each sampled 4 times. Pigs for blood sampling within the pens were selected as every n^th^ pig in the pen depending on the number of pigs per pen, assuring that 5 or 4 pigs per pen were selected in Herd 1 and 2, respectively. In Herd 2, the same 4 pigs per pen were bled at the 4 consecutive sampling time points (unless death or early removal had occurred, in which case a substitute pig was randomly selected).

### Blood sampling

Blood samples were collected from the cranial vena cava in plain tubes. In Herd 1, the blood samples were transported directly to the laboratory after sampling and refrigerated overnight. In Herd 2, samples were kept refrigerated overnight and were shipped to the laboratory by mail the following day.

At the laboratory, blood samples were centrifuged to separate serum. Equal amounts of serum from each of the 4/5 pigs per pen were pooled prior to PCV2-qPCR analysis, resulting in one serum PCV2 copy number (viral load) per pen. Thus, serum viral loads refer to qPCR-results from pooled serum samples.

### Oral fluid sampling

Sampling of oral fluid was performed as previously described [19]. Briefly, a cotton rope, fixed to the pen railings, was presented to the pigs allowing them to chew on it, thereby transferring oral fluid to the rope. Thirty min later, ropes were collected in individual plastic bags and wringed to release the oral fluid for later qPCR-analysis. Based on numerous observations during sampling, it was estimated that more than 80% of the pigs in a pen contributed to the oral fluid sample. Storage and shipment of oral fluid samples to the laboratory were as described for the blood samples.

### Quantification of PCV2 in oral fluid and serum by qPCR

The oral fluids collected in both herds were grey and dirty in appearance, probably reflecting fecal contamination. Feces as well as saliva can contain PCR inhibitors [34–36] and if these are present during qPCR-analysis, underestimation of quantitative levels or false-negative results may occur. To eliminate the effect of potential PCR inhibitors in oral fluid, the samples were centrifuged and diluted 1:10 in nuclease-free water^1^ prior to DNA extraction. The applicability of this pre-extraction dilution was confirmed by testing 5 naturally PCV2-positive oral fluid samples and 5, initially PCV2-negative, oral fluid samples spiked 1:100 with PCV2 virus isolate. Evaluation was performed by comparison of Ct-values and PCR efficiencies calculated from 10-fold dilution series of extracted DNA and tested for PCV2 as described below.

DNA extraction from all oral fluid samples and the serum samples from Herd 2 was performed with a commercially available extraction kit^2^ using 200 μl serum or 200 μl oral fluid diluted 1:10 in nuclease free water^1^. DNA extraction from serum from Herd 1 was performed differently^3^ but internal laboratory validation was performed by testing 78 samples using both methods. On average, the results obtained by the two methods were very similar with an average difference of 0.2 log(10) PCV2 copies per ml serum (range 0.0–1.6).

The serum and oral fluid samples were tested for PCV2-DNA by qPCR essentially as previously described [37]. During the testing of some of the oral fluid samples from Herd 2 an inhibition of the qPCR was revealed, probably due to a fava bean feed ingredient. Therefore, the DNA extracted from these samples were tested both undiluted and diluted 1:10 in nuclease-free water^1^ to avoid false negative test results. Oral fluid viral loads were subsequently corrected according to the extra dilutions. The qPCR-assay had a detection limit of 10^3^ and a quantification range of 3.3 × 10^4^–3.3 × 10^9^ PCV2 copies per ml [37]. Because the oral fluid was diluted 10 times prior to DNA extraction, the minimum concentration that could be detected in the samples was 10 times higher for OF compared to serum (10^4^ versus 10^3^). Samples were considered positive when the viral load was above the detection limit.

### Statistical analyses

All PCV2 viral loads were analyzed on a log-transformed scale. Comparison of PCV2 viral loads in serum and oral fluid was made separately for Herd 1 and 2, and because the same pens were repeatedly sampled in Herd 2, and hence could not be considered independent, each sampling time point in Herd 2 was also analyzed separately. Viral loads below the assay detection limit were included in the statistical analyses with a value of 0, since the true distribution of these was unknown and excluding the observations would reduce the actual variation and thereby bias the results.

Descriptive statistics consisted of frequency distributions, graphical illustrations and summary statistics. Evaluation of agreement between serum and oral fluid PCV2 viral loads was done both on a dichotomous (PCV2-positive/negative) and a quantitative scale. On a dichotomous scale, the oral fluid cut-off value for obtaining the best agreement with a PCV2-positive serum result (above the test detection limit of 3 log(10) PCV2-copies per ml serum) was estimated by drawing a receiver operating characteristic (ROC)-curve for all possible oral fluid cut-off values against a serum value fixed at the assay detection limit. Best agreement was defined as the oral fluid cut-off value where relative sensitivity and relative specificity were maximized simultaneously. The terms ‘relative sensitivity’ and ‘relative specificity’ were used, since the serum result could not be considered ‘gold standard’ in the classical sense but rather a ‘reference standard’ (as in a study from 2003 [38]). All sample pairs (serum and oral fluid collected from the same pen) in Herd 1 and sample pairs from the first sampling in Herd 2 were used for this purpose, since only those included more than one PCV2-negative serum result. On a quantitative scale, the viral loads in serum and oral fluid were compared and the correlation coefficients estimated. Due to the non-normal distribution of serum and oral fluid PCV2 viral loads, non-parametric tests were used (paired Wilcoxon-test and Spearman’s rank correlation test). All statistical analyses were performed in R [39] with a significance level set at 0.05. However, due to multiple comparisons, the significance level was adjusted by the Bonferroni method. Finally, in order to evaluate an eventual effect of the number of pigs per pen at sampling on the oral fluid viral loads, two linear regressions with oral fluid viral load as the outcome were performed, one for each herd. For Herd 1, serum pool viral load and number of pigs per pen were included as explanatory variables. For Herd 2, also sampling number and the interaction between pigs per pen at sampling and sampling number were included as additional explanatory variables. Model selection was based on a backwards elimination procedure, also with a significance level of 0.05 for keeping variables in the models. The final models´ distribution of residuals was assessed visually for normality.

## Results

In total, 310 serum and oral fluid sample pairs were collected. Of these, 50 sample pairs were from Herd 1 with 4–5 pigs bled per pen with a mean of 23 pigs per pen (range: 5–33) and 260 sample pairs (65 pens sampled 4 times) were from Herd 2 with 4 pigs bled per pen with a mean of 29 pigs per pen (range: 9–32). The number of pigs per pen reflects the maximum number of pigs contributing to the oral fluid sample.

### Agreement between serum pools and oral fluid for classification of pens into PCV2 positive/negative

Classification of sample pairs into PCV2 positive and PCV2 negative based on the test detection limit is shown in Table 1. In Herd 1, 80% of serum pools and 86% of oral fluid samples were PCV2 positive, whereas totally in Herd 2, 91% of serum pools and 100% of oral fluid samples were PCV2 positive. Of the 23 negative serum pools in Herd 2, 22 were negative at the first sampling.

**Table 1:** Distribution of serum and oral fluid samples below (negative) versus above (positive) the PCV2-qPCR test detection limit for finishing pigs in 2 herds

				Serum	
	Sampling			Positive	Negative
Herd 1 (*n* = 50)	1	Oral fluid	Positive	39	4
			Negative	1	6
Herd 2 (*n* = 65)	1	Oral fluid	Positive	43	22
			Negative	0	0
	2	Oral fluid	Positive	65	0
			Negative	0	0
	3	Oral fluid	Positive	64	1
			Negative	0	0
	4	Oral fluid	Positive	65	0
			Negative	0	0
Total				277	33

**Fig. 1:**
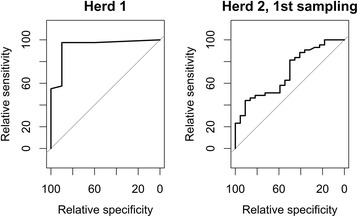
ROC curves from Herd 1 (left) and first sampling in Herd 2 (right) to estimate the oral fluid cut-off for obtaining the best agreement with a PCV2-positive serum pool result for finishing pigs

The 2 ROC-curves (one for Herd 1 and one for the first sampling in Herd 2) for evaluation of best agreement between serum and oral fluid concerning a PCV2-positive serum result are displayed in Fig. 1.The cut-off value for oral fluid associated with the best agreement with a PCV2-positive serum result was estimated to be 6.5 and 7.36 log(10) PCV2 copies per ml serum for Herd 1 and 2, respectively (Table 2).

**Table 2:** Oral fluid cut-off value best agreeing with a PCV2-positive serum pool in finishers

Best agreement	Oral fluid cut-off (log(10))	Relative sensitivity (95% C.I.)	Relative specificity (95% C.I.)	Area under curve (95% C.I.)
Herd 1	6.50	0.98 (0.87;1.00)	0.90 (0.55;1.00)	0.941 (0.851;1.00)
Herd 2, 1st sampling	7.36	0.58 (0.42;0.73)	0.59 (0.36;0.79)	0.7 (0.567; 0.833)

### Comparison of serum and oral fluid PCV2 viral loads

**Fig. 2:**
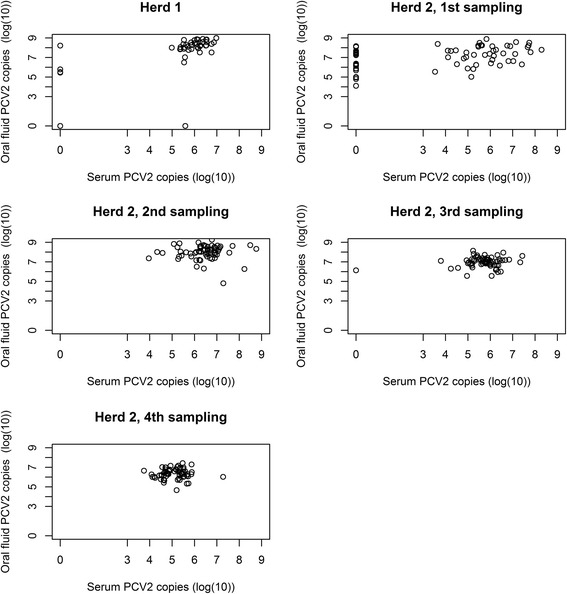
Plots of serum pools and oral fluid sample pairs from finishing pigs for both herds and all samplings with serum pool viral loads on the x-axis and oral fluid viral loads on the y-axis

**Fig. 3:**
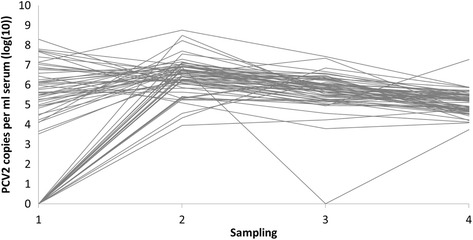
Evolution with time in Herd 2 of PCV2 viral loads in serum pools. Samplings were done at 3-week intervals and each line represents one pen

**Fig. 4:**
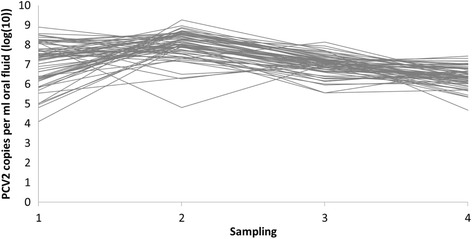
Evolution with time in Herd 2 of PCV2 viral loads in oral fluid. Samplings were done at 3-week intervals and each line represents one pen

Figure 2 contains plots of the serum and oral fluid sample pairs from both herds at each sampling time point. As the sampling in Herd 2 was longitudinal, the evolution with time of serum and oral fluid viral loads are additionally shown in Figs. 3 and 4, respectively. Summary statistics of and estimated Spearman’s correlation coefficients between sample pairs by herd and sampling time point are displayed in Table 3. A pairwise comparison of the quantitative viral loads in serum and oral fluid showed a significantly higher number of PCV2 copies in oral fluid compared to serum for both herds and at all sampling time points. The overall mean differences between oral fluid and serum were 2.10 log(10) (1Q,3Q = 1.68, 2.63) and 1.83 (1Q,3Q = 0.88, 2.11) for Herd 1 and 2, respectively. For Herd 1 and the first sampling in Herd 2, significant correlations between serum and oral fluid were found. However, no correlations were found for samplings 2, 3 and 4 in Herd 2.

**Table 3:** Summary statistics of PCV2 viral loads and estimated Spearman’s correlation coefficients (ρ) between oral fluid and serum pools from two finishing herds

PCV2 viral load	Sampling no.	Sample type	Mean	Median	Standard deviation	Min	Max	Wilcoxon *(p*-value)^c^	Spearman’s correlation	
									ρ	*p*-value^c^
Herd 1	1	Serum^a^	4.82	5.86	2.47	0	6.97	<0.001	0.69	<0.001
(*n* = 50)		OF^b^	6.92	8.13	2.92	0	8.99			
Herd 2	1	Serum	3.89	4.99	2.96	0	8.29	<0.001	0.39	0.001
(*n* = 65)		OF	7.09	7.37	1.07	4.1	8.89			
	2	Serum	6.43	6.51	0.86	3.96	8.76	<0.001	0.14	0.278
		OF	7.98	8.03	0.73	4.80	9.26			
	3	Serum	5.69	5.79	0.96	0	7.43	<0.001	0.04	0.725
		OF	6.97	7.03	0.51	5.56	8.13			
	4	Serum	5.09	5.19	0.56	3.74	7.28	<0.001	0.08	0.524
		OF	6.37	6.37	0.51	4.67	7.43			

^a^Serum = serum pool, ^b^OF = oral fluid, ^c^significance level is 0.005 due to Bonferroni correction

Results from the linear regression models showed that oral fluid viral load was not significantly influenced by the number of pigs per pen when the viral load in serum (Herd 1 and 2) and sampling number (Herd 2) were also included in the models.

## Discussion

Overall, both herds had a widespread PCV2 infection with moderate viral loads resulting in relatively few serum pools and even fewer oral fluid samples being negative. A relatively small number of serum pools had viral loads above 7 log(10), which fits well with the observation that no clinical signs clearly related to PCV2 infection were present in either of the 2 herds. This, however, also implies that generalizing results to herds with high viral loads and/or clinical signs of PCV2 infection should be done with caution. Only one sample pair had a positive serum PCV2 result (5.59 log(10) PCV2 copies per ml serum) and a negative oral fluid PCV2 result, which may reflect that not all blood-sampled pigs chewed on the rope. The estimated contribution to the oral fluid sample of more than 80% of the pigs in a pen is supported by a study reporting that 94% of pigs in a pen (pen size: 17–24 pigs) had chewed on the presented rope after 30 min [40].

As expected, a slightly higher proportion of oral fluid samples compared to serum were PCV2 positive which probably reflects that the likelihood of a positive result is higher when more animals are tested (4 or 5 pigs were bled versus up to 33 pigs contributing to the oral fluid sample). This is similar to previous findings concerning PCV2 [24, 29] and more general findings reporting increasing herd sensitivities at increasing pool sizes [41]. Alternatively, contamination of the rope with PCV2 present in feces could also explain the difference, as a previous study has shown a higher proportion of positive rectal swabs compared to serum samples from individual animals [9]. In Herd 2, this difference in positive proportions was only evident at the first sampling with 66% positive serum pools versus 100% positive oral fluid samples, probably reflecting a lower within-pen prevalence of PCV2 compared to the subsequent samplings, when the infection may have spread. Based on simple sample size calculations, with a 95% probability of finding at least one positive animal, 4 negative samples can be achieved even with a 50% disease prevalence, whereas a sample size of 20 (approximation of the pigs sampled by oral fluid) would detect disease at a prevalence below 10% [42]. Consequently, the risk of overlooking a PCV2 infection, when the within-pen prevalence is low, seems increased if serum samples from a few pigs, instead of oral fluid samples from many pigs, are used.

The estimated oral fluid cut-offs for best agreement (the highest possible relative sensitivity and specificity) with a PCV2-positive serum pool were fairly similar for Herd 1 and 2 (first sampling) with 6.5 and 7.36 log(10) PCV2 copies per ml oral fluid, respectively. However, the oral fluid cut-off was determined with a higher accuracy in terms of relative sensitivity and specificity for Herd 1 than for Herd 2, probably due to the lower variation between individual oral fluid results in Herd 1 compared to Herd 2 (first sampling) (see Fig. 2). This has previously been described as a well-known challenge in diagnostic test evaluation [43].

The viral loads in oral fluid were significantly higher compared to the matched serum pools. A difference of almost 2 log(10) PCV2 copies per ml sample was found in both herds, hence, substantially higher than the 1 log(10) reported by others [29, 30]. Whether this divergence is merely due to PCR-assay differences is unknown. As with the higher proportion of positives, a higher viral load in oral fluid was expected because far more pigs were sampled with oral fluid. Earlier studies demonstrating a high variation of viral loads in serum between individual pigs within a group support this [18, 44]: In 10 animals in each of 5 different PMWS-negative farms, ranges between <4 (detection limit) and 8.7 log(10) PCV2 copies per ml serum within the same farm and age group were found [18]. And in a vaccination trial, a mean level of 6.1 log(10) and a standard deviation of 1.7 log(10) PCV2 copies per ml serum in 8 PCV2-positive control pigs were reported [44]. Consequently, the specific viral load found in a positive serum pool is prone to vary greatly, being very dependent upon which pigs happen to be selected for blood sampling as opposed to oral fluid sampling that includes nearly all pigs in a pen. The results from Herd 2 nicely demonstrate this when comparing Figs. 3 and 4 which show the evolution with time. Here, a higher variation between individual-pen serum viral loads compared to individual-pen oral fluid viral loads is generally seen.

Secondly, higher PCV2 viral loads may be present in oral fluid compared to the viral loads found in serum. Previous comparisons of serum and oral/tonsillar swabs found either no difference concerning prevalence of PCV2 positives [6, 45] and viral loads [6, 7] or a higher prevalence [7] and higher viral loads [45] in oral/tonsillar swabs compared to serum. Furthermore, in Herd 1, 2 pens contained only 5 pigs resulting in 100% of pigs being blood sampled. Still, the oral fluid PCV2 viral loads were higher than the serum viral loads with 8.07 and 7.03 log(10) in oral fluid versus 6.12 and 5.57 log(10) in serum.

A significant correlation between serum and oral fluid viral loads was found for Herd 1 and the first sampling in Herd 2, whereas no significant correlations were found for samplings 2, 3 and 4 in Herd 2. For Herd 1, the estimated correlation coefficient of 0.69 was comparable to the 0.76 and 0.78 previously reported [24, 29]. However, of these previously reported correlation coefficients, only the coefficient of 0.78 was found to be significant [24]. This was based on 18 pens sampled 5 consecutive times thus challenging the assumption of independency between observations and an analysis of time points individually, as in the current study, might have given a different result.

For the first sampling in Herd 2, the correlation coefficient, even though significant, was only estimated to 0.39, which may be due to the high number of negative serum pools. For samplings 2, 3 and 4 in Herd 2, no statistically significant correlations were found between serum pools and oral fluid. A higher proportion of pigs in the pen were blood sampled in Herd 1 (~ 20%) compared to Herd 2 (~14%), which would seem like a plausible explanation, but the results from the linear regression models showed that the number of pigs per pen at sampling did not influence the oral fluid viral load when the serum viral load (Herd 1 and 2) and sampling number (Herd 2) were accounted for. Another explanation could be the higher variation in positive serum viral loads for all samplings in Herd 2 compared to Herd 1 (Fig. 2) which could reduce the likelihood of finding a significant correlation between serum and oral fluid, if it existed. Nevertheless, with the datasets included in this study, a cut-off for oral fluid corresponding to the established cut-off regarding clinical signs for serum of 7 log(10) PCV2 copies per ml serum [8–10] could not be determined.

## Conclusions

In conclusion, a slightly higher proportion of PCV2 positive pens and higher viral loads were found with oral fluid as sample material compared to serum pools. Furthermore, oral fluid seemed to detect a PCV2 infection earlier with viral loads as high as 7 log(10) being associated with a negative serum pool for the same pen. Nevertheless, a statistically significant correlation between serum pools and oral fluid samples could not be found for all sampling time points, probably due to a high within-pen variation in individual pig viral load becoming very evident in serum pools including only four or five pigs in a pen of around 30. Hence, from a practitioner’s point of view, oral fluid might be better suited to identify presence or not of the pathogen than to determine the specific viral load.

## Footnotes

1 UltraPure™ DNase/RNase-Free Distilled Water, Invitrogen, Nærum, Denmark.

2 QIAamp DNA Mini Kit automated on QIAcube extraction robot, QIAGEN, Copenhagen, Denmark.

3 QIAsymphony Virus/Bacteria Mini Kit, protocol Pathogen complex 200 without IC V2, with elution in 110 μl elution buffer, automated on QIASymphony extraction robot, QIAGEN, Copenhagen, Denmark.
